# Supplementary material for: A systematic review on the relationship between the built environment and children’s quality of life
Source: BMC Public Health. 2023 Dec 11;23:2472. doi: 10.1186/s12889-023-17388-8 (PMC10714453; doi:10.1186/s12889-023-17388-8)
Supplement: Supplementary file 1 — Additional file 1: Appendix A. Tailored full search strings for databases. [file 12889_2023_17388_MOESM1_ESM.docx]

**Appendices**

**Appendix A – Tailored full search strings for databases**

**PubMed**

("quality of life"[MeSH Terms] OR QOL[Title/Abstract] OR HRQOL[Title/Abstract] OR "health-related quality of life"[Title/Abstract] OR wellbeing[Title/Abstract] OR “well-being”[Title/Abstract] OR “well being”[Title/Abstract])

AND

(pediatric[Title/Abstract] OR child*[Title/Abstract] OR adolescent*[Title/Abstract] OR kids[Title/Abstract] OR "young people"[Title/Abstract] OR girl*[Title/Abstract] OR boys[Title/Abstract] OR teenager*[Title/Abstract] OR pupil*[Title/Abstract] OR youth*[Title/Abstract] OR student*[Title/Abstract])

AND

("built environment*"[MeSH Terms] OR "physical environment*"[Title/Abstract] OR "living environment*"[Title/Abstract] OR "urban environment*"[Title/Abstract] OR "rural environment*"[Title/Abstract] OR "environment design"[MeSH Terms] OR "urban design"[Title/Abstract] OR "environment* feature*"[Title/Abstract] OR "environment* characteristic*"[Title/Abstract] OR "community feature*"[Title/Abstract] OR "community characteristic*"[Title/Abstract] OR "natural environment*"[Title/Abstract] OR nature[Title/Abstract] OR "open space*"[Title/Abstract] OR "parks, recreational"[MeSH Terms] OR "public space*"[Title/Abstract] OR playground*[Title/Abstract] OR "green space*"[Title/Abstract] OR greenspace*[Title/Abstract] OR "urban form*"[Title/Abstract] OR traffic[Title/Abstract] OR neighborhood[Title/Abstract] OR neighbourhood[Title/Abstract] OR "living condition*"[Title/Abstract] OR "environmental condition*"[Title/Abstract])

**CINAHL & PsycInfo**

("quality of life" OR QOL OR HRQOL OR "health-related quality of life" OR wellbeing OR “well-being” OR “well being”)

AND

(pediatric OR child* OR adolescent* OR kids OR "young people" OR girl* OR boys OR teenager* OR pupil* OR youth* OR student*)

AND

("built environment*" OR "physical environment*" OR "living environment*" OR "urban environment*" OR "rural environment*" OR "environment design" OR "urban design" OR "environment* feature*" OR "environment* characteristic*" OR "community feature*" OR "community characteristic*" OR "natural environment*" OR nature OR "open space*" OR parks OR "public space*" OR playground* OR "green space*" OR greenspace* OR "urban form*" OR traffic OR neighborhood OR neighbourhood OR "living condition*" OR "environmental condition*")

**Embase**

('quality of life':ab,ti OR qol:ab,ti OR hrqol:ab,ti OR 'health-related quality of life':ab,ti OR wellbeing:ab,ti OR ‘well-being’:ab,ti OR ‘well being’:ab,ti)

AND

(pediatric:ab,ti OR child*:ab,ti OR adolescent*:ab,ti OR kids:ab,ti OR 'young people':ab,ti OR girl*:ab,ti OR boys:ab,ti OR teenager*:ab,ti OR pupil*:ab,ti OR youth*:ab,ti OR student*:ab,ti)

AND

('built environment*':ab,ti OR 'physical environment*':ab,ti OR 'living environment*':ab,ti OR 'urban environment*':ab,ti OR 'rural environment*':ab,ti OR 'environment design':ab,ti OR 'urban design':ab,ti OR 'environment* feature*':ab,ti OR 'environment* characteristic*':ab,ti OR 'community feature*':ab,ti OR 'community characteristic*':ab,ti OR 'natural environment*':ab,ti OR nature:ab,ti OR 'open space*':ab,ti OR parks:ab,ti OR 'public space*':ab,ti OR playground*:ab,ti OR 'green space*':ab,ti OR greenspace*:ab,ti OR 'urban form*':ab,ti OR traffic:ab,ti OR neighborhood:ab,ti OR neighbourhood:ab,ti OR 'living condition*':ab,ti OR 'environmental condition*':ab,ti) AND [2000-2021]/py

AND

([adolescent]/lim OR [preschool]/lim OR [school]/lim)

AND

([english]/lim OR [german]/lim)

**Web of Science**

(TS=("quality of life") OR TS=(QOL) OR TS=(HRQOL) OR TS=("health-related quality of life") OR TS=(wellbeing) OR TS=("well-being") OR TS=("well being"))

AND
(TS=(pediatric) OR TS=(child*) OR TS=(adolescent*) OR TS=(kids) OR TS=("young people") OR TS=(girl*) OR TS=(boys) OR TS=(teenager*) OR TS=(pupil*) OR TS=(youth*) OR TS=(student*))

AND

(TS=("built environment*") OR TS=("physical environment*") OR TS=("living environment*") OR TS=("urban environment*") OR TS=("rural environment*") OR TS=("environment design") OR TS=("urban design") OR TS=("environment* feature*") OR TS=("environment* characteristic*") OR TS=("community feature*") OR TS=("community characteristic*") OR TS=("natural environment*") OR TS=(nature) OR TS=("open space*") OR TS=(parks) OR TS=("public space*") OR TS=(playground*) OR TS=("green space*") OR TS=(greenspace*) OR TS=("urban form*") OR TS=(traffic) OR TS=(neighborhood) OR TS=(neighbourhood) OR TS=("living condition*") OR TS=("environmental condition*"))
