# Supplementary material for: A systematic review on the relationship between the built environment and children’s quality of life
Source: BMC Public Health. 2023 Dec 11;23:2472. doi: 10.1186/s12889-023-17388-8 (PMC10714453; doi:10.1186/s12889-023-17388-8)
Supplement: Supplementary file 2 — Additional file 2: Supplemental Material Table 1 (S1). Reasons for exclusion during title/abstract screening. [file 12889_2023_17388_MOESM2_ESM.docx]

**Supplemental Material Table 1 (S1): Reasons for exclusion during title/abstract screening**

| **Start Title/Abstract Screening** | **4,287** |
| --- | --- |
| Population does not meet inclusion criteria | 879 |
| Clinical topics, disease/disorder-related, clinical population | 1,455 |
| Study protocols | 53 |
| Conference proceeding/annual meeting descriptions | 8 |
| Validation, development or feasibility studies, translations, evaluation of screening tools or questionnaires, framework or guidelines | 120 |
| (Systematic) reviews, meta-analyses, commentaries, perspective pieces, editorials, methodological studies, qualitative studies | 171 |
| Exposure (built environment) does not meet inclusion criteria | 910 |
| Outcome (pediatric QOL) did not meet inclusion criteria | 624 |
| Full text not available | 1 |
| Other (historical analysis, books, corrigendums, factor analysis, film analysis, lecture, literature analysis, non-human subjects) | 17 |
| **End Title/Abstract Screening** | **49** |
