# Supplementary material for: A systematic review on the relationship between the built environment and children’s quality of life
Source: BMC Public Health. 2023 Dec 11;23:2472. doi: 10.1186/s12889-023-17388-8 (PMC10714453; doi:10.1186/s12889-023-17388-8)
Supplement: Supplementary file 3 — Additional file 3: Supplemental Material Table 2 (S2). Reasons for exclusion during full-text screening. [file 12889_2023_17388_MOESM3_ESM.docx]

**Supplemental Material Table 2 (S2): Reasons for exclusion during full-text screening**

| **Start Full-text screening** | **49** |
| --- | --- |
| Built environment not assessed | 15 |
| Mixed age groups involving adults | 3 |
| QoL/wellbeing not assessed | 12 |
| QoL/built environment not analyzed together | 2 |
| **End Full-text screening** | **17** |
