# Supplementary material for: A systematic review on the relationship between the built environment and children’s quality of life
Source: BMC Public Health. 2023 Dec 11;23:2472. doi: 10.1186/s12889-023-17388-8 (PMC10714453; doi:10.1186/s12889-023-17388-8)
Supplement: Supplementary file 4 — Additional file 4: Supplemental Material Table 3 (S3). Study-level quality of evidence for included studies. [file 12889_2023_17388_MOESM4_ESM.docx]

***Supplemental Material Table 3 (S3): Study-level quality of evidence for included studies***

| Cross-Sectional Studies | | | | | | | | | |
| --- | --- | --- | --- | --- | --- | --- | --- | --- | --- |
| Reference | Were the criteria for inclusion in the sample clearly defined? | Were the study subjects and the setting described in detail? | Was the exposure measured in a valid and reliable way? | Were objective, standard criteria used for measurement of the condition? | Were confounding factors identified? | Were strategies to deal with confounding factors stated? | Were the outcomes measured in a valid and reliable way? | Was appropriate statistical analysis used? | Study-level quality of evidence* |
| Kim, J. H., et al. (2016)  Reference number: [26] | ✓ | ✘ | ✓ | ✓ | ✓ | ✓ | ✓ | ✓ | Good |
| Martin, G., et al. (2021)  Reference number: [27] | ✓ | ✓ | ✓ | ✓ | ✓ | ✓ | ✓ | ✓ | Good |
| McCracken, D. S., et al. (2016)  Reference number: [28] | ✓ | ✓ | ✓ | ✓ | ✓ | ✓ | ✓ | ✓ | Good |
| Tillmann, S., et al. (2018)  Reference number: [29] | ✓ | ✓ | ✓ | ✓ | ✓ | ✘ | ✓ | ✓ | Good |
| Weigl, K., et al. (2018)  Reference number: [30] | ✘ | ✓ | ✓ | ✓ | ✓ | ✓ | ✓ | ✓ | Good |
| Wu, X. Y., et al. (2010)  Reference number: [31] | ✓ | ✓ | ✓ | ✓ | ✓ | ✓ | ✓ | ✓ | Good |
| Nagata et al. (2021)  Reference number: [33] | ✓ | ✓ | ✓ | ✓ | ✓ | ✓ | ✓ | ✓ | Good |
| González-Carrasco et al. (2019)  Reference number: [35] | ✘ | ✓ | ✓ | ✓ | ✓ | ✘ | ✓ | ✓ | Fair |
| Nordbø et al. (2020)  Reference number: [36] | ✓ | ✓ | ✓ | ✓ | ✓ | ✓ | ✓ | ✓ | Good |
| de Macêdo et al. (2021)  Reference number: [37] | ✘ | ✓ | ✓ | ✓ | ✓ | ✓ | ✓ | ✘ | Fair |
| Lee & Yoo (2015)  Reference number: [38] | ✓ | ✓ | ✓ | ✓ | ✓ | ✓ | ✓ | ✓ | Good |
| de Bont et al. (2021)  Reference number: [25] | ✓ | ✓ | ✓ | ✓ | ✓ | ✓ | ✓ | ✓ | Good |
| Mitra et al. (2021)  Reference number: [40] | ✓ | ✓ | ✓ | ✓ | ✓ | ✓ | ✓ | ✓ | Good |
| Forrester et al. (2022)  Reference number: [41] | ✓ | ✓ | ✓ | ✓ | ✓ | ✓ | ✓ | ✓ | Good |

| Quasi-Experimental/Intervention Studies | | | | | | | | | | |
| --- | --- | --- | --- | --- | --- | --- | --- | --- | --- | --- |
| Reference | Is it clear in the study what is the ‘cause’ and what is the ‘effect’ (i.e. there is no confusion about which variable comes first)? | Were the participants included in any comparisons similar? | Were the participants included in any comparisons receiving similar treatment/care, other than the exposure or intervention of interest? | Was there a control group? | Were there multiple measurements of the outcome both pre and post the intervention/exposure? | Was follow up complete and if not, were differences between groups in terms of their follow up adequately described and analyzed? | Were the outcomes of participants included in any comparisons measured in the same way? | Were outcomes measured in a reliable way? | Was appropriate statistical analysis used? | Study-level quality of evidence* |
| Wallner et al. (2018)  Reference number: [39] | ✓ | ✓ | ✓ | ✓ | ✓ | ✓ | ✓ | ✓ | ✓ | Good |

| Longitudinal/Cohort Studies | | | | | | | | | | | | |
| --- | --- | --- | --- | --- | --- | --- | --- | --- | --- | --- | --- | --- |
| Reference | Were the two groups similar and recruited from the same population? | Were the exposures measured similarly to assign people to both exposed and unexposed groups? | Was the exposure measured in a valid and reliable way? | Were confounding factors identified? | Were strategies to deal with confounding factors stated? | Were the groups/ participants free of the outcome at the start of the study (or at the moment of exposure)? | Were the outcomes measured in a valid and reliable way? | Was the follow up time reported and sufficient to be long enough for outcomes to occur? | Was follow up complete, and if not, were the reasons to loss to follow up described and explored? | Were strategies to address incomplete follow up utilized? | Was appropriate statistical analysis used? | Study-level quality of evidence* |
| Feng et al. (2017)  Reference number: [34] | N/A | N/A | ✓ | ✓ | ✓ | ✓ | ✓ | ✓ | ✘ | ✘ | ✓ | Fair |
| Mastorci, F., et al. (2021)  Reference number: [32] | ✓ | ✓ | ✓ | ✓ | ✓ | ✓ | ✓ | ✓ | ✘ | N/A | ✓ | Good |

* Study-level quality of evidence was assessed with the Joanna Briggs Institute Critical Appraisal Checklists. The proper checklist was chosen according to study type. <2 items marked as “no” or “undetermined” (✘) qualified for good methodological quality, 2-3 items marked as “no” or “undetermined” (✘) qualified as fair methodological quality, and >3 items marked as “no” or “undetermined” qualified (✘) as poor methodological quality.

**References**

20. de Bont, J., Márquez, S., Fernández-Barrés, S., Warembourg, C., Koch, S., Persavento, C., Fochs, S., Pey, N., de Castro, M., & Fossati, S. (2021). Urban environment and obesity and weight-related behaviours in primary school children. Environ Int, 155, 106700.

21. Kim, J. H., Lee, C., & Sohn, W. (2016). Urban natural environments, obesity, and health-related quality of life among hispanic children living in inner-city neighborhoods. Int J Environ Res Public Health, 13(1).

22. Martin, G., Graat, M., Medeiros, A., Clark, A. F., Button, B. L. G., Ferguson, K. N., & Gilliland, J. A. (2021). Perceived neighbourhood safety moderates the relationship between active school travel and health-related quality of life. Health Place, 70, 102623.

23. McCracken, D. S., Allen, D. A., & Gow, A. J. (2016). Associations between urban greenspace and health-related quality of life in children. Preventive medicine reports, 3, 211-221.

24. Tillmann, S., Clark, A. F., & Gilliland, J. A. (2018). Children and nature: Linking accessibility of natural environments and children’s health-related quality of life. Int J Environ Res Public Health, 15(6), 1072.

25. Weigl, K., Herr, C. E. W., Meyer, N., Otto, C., Stilianakis, N., Bolte, G., Nennstiel-Ratzel, U., & Kolb, S. (2018). Prädiktoren gesundheitsbezogener Lebensqualität bei bayerischen Einschulungskindern. Gesundheitswesen, 80(S 01), S1-S4.

26. Wu, X. Y., Ohinmaa, A., & Veugelers, P. J. (2010). Sociodemographic and neighbourhood determinants of health-related quality of life among grade-five students in Canada. Qual Life Res, 19(7), 969-976.

27. Mastorci, F., Piaggi, P., Doveri, C., Trivellini, G., Casu, A., Pozzi, M., Vassalle, C., & Pingitore, A. (2021). Health-related quality of life in italian adolescents during covid-19 outbreak. Front Pediatr, 9(129).

28. Nagata, M., & Liehr, P. (2021). Urban children’s well-being factors and qualities of being and doing in natural space: Nature immersion. J Holist Nurs, 39(2), 174-184.

29. Feng, X., & Astell-Burt, T. (2017). Residential green space quantity and quality and child well-being: a longitudinal study. Am J Prev Med, 53(5), 616-624.

30. González-Carrasco, M., Casas, F., Ben-Arieh, A., Savahl, S., & Tiliouine, H. (2019). Children’s perspectives and evaluations of safety in diverse settings and their subjective well-being: A multi-national approach. Appl Res Qual Life, 14(2), 309-334.

31. Nordbø, E. C. A., Raanaas, R. K., Nordh, H., & Aamodt, G. (2020). Disentangling how the built environment relates to children's well-being: Participation in leisure activities as a mediating pathway among 8-year-olds based on the Norwegian mother and child cohort study. Health Place, 64, 102360.

32. de Macêdo, C. M. V., Gil, M., & Strelhow, M. R. W. (2022). Urban mobility and subjective well-being among brazilian children. Child Indic Res, 15(2), 467-485.

33. Lee, B. J., & Yoo, M. S. (2015). Family, school, and community correlates of children’s subjective well-being: An international comparative study. Child Indic Res, 8(1), 151-175.

34. Wallner, P., Kundi, M., Arnberger, A., Eder, R., Allex, B., Weitensfelder, L., & Hutter, H.-P. (2018). Reloading pupils’ batteries: Impact of green spaces on cognition and wellbeing. Int J Environ Res Public Health, 15(6), 1205.

35. Mitra, R., Waygood, E. O. D., & Fullan, J. (2021). Subjective well-being of Canadian children and youth during the COVID-19 pandemic: The role of the social and physical environment and healthy movement behaviours. Preventive Medicine Reports, 23, 101404.

36. Forrester, P., Kahric, U., Lewis, E. M., & Rose, T. (2022). Family, Peer, and Neighborhood Influences on Urban Children’s Subjective Wellbeing. Child Adolesc Social Work J, 1-13.
